# Unraveling migraine susceptibility in females: the involvement of GABA genes

**DOI:** 10.1186/1129-2377-14-S1-P11

**Published:** 2013-02-21

**Authors:** MS Quintas, JL Neto, J Pereira-Monteiro, J Barros, J Sequeiros, A Sousa, I Alonso, C Lemos

**Affiliations:** 1 IBMC, Portugal; 2 ICBAS, Portugal

## 

Migraine is a common neurological disorder with a global prevalence around 10% [1]. Several studies showed that migraine is influenced by genetic and environmental factors [2]. The female-to-male ratio of migraine prevalence is 3- to 4-fold higher among women than men[3]. The role of common variants of GABA genes in the X-chromosome in migraine susceptibility was assessed, aiming to explain the differences in disease frequency between males and females. An association study with 188 unrelated cases and 287 migraine-free controls age- and ethnic matched was performed. The case-control ratio was 1:1.5. Candidate genes were selected based on their possible role in pathophysiology of migraine.Thirty-two tagging SNPs were selected in three genes (GABRE, GABRA3 and GABRQ) and genotyping was performed by SNaPshot. Allelic, genotypic and haplotypic frequencies were compared between cases and controls and multiple testing corrections were performed. Also, gene-gene interactions were analyzed. The Results for allelic associations revealed five nominal significant associations and three trends for association. In what concerns genotypic frequencies, four noteworthy results were found in GABRE and GABRA3 genes. After multiple testing correction, two allelic associations remained significant (GABRA3 and GABRE) and one genotypic association resisted to Bonferroni correction (GABRE), all in the females group. No significant results were found in the haplotypic analyses but an additive effect was observed between two SNPs of GABRA3 and two of GABRE. These findings show, for the first time, evidence of a possible involvement of common variants in GABA receptors in migraine susceptibility and in gender-specific liability.
